# Evidence for Chronic Kidney Disease-Mineral and Bone Disorder Associated With Metabolic Pathway Changes

**DOI:** 10.1097/MD.0000000000001273

**Published:** 2015-08-14

**Authors:** Qiong Wu, Xueli Lai, Zhenyu Zhu, Zhanying Hong, Xin Dong, Tieyun Wang, Haiyan Wang, Ziyang Lou, Qishan Lin, Zhiyong Guo, Yifeng Chai

**Affiliations:** From the School of Pharmacy (QW, ZZ, ZH, XD, ZL, YC); Department of Nephrology (XL, TW, HW, ZG), Changhai Hospital, Second Military Medical University, Shanghai, China; and Proteomics/Mass Spectrometry Facility (QL), Center for Functional Genomics, University at Albany, Rensselaer, NY.

## Abstract

Abnormalities in the levels of calcium, phosphorus, and parathyroid hormone (PTH) in serum are typical for patients with chronic kidney disease (CKD). They are used routinely to predict the onset of CKD-mineral and bone disorder (MBD). However, CKD-MBD associated with metabolic pathway imbalance is not well understood.

The objective of the study was to identify endogenous metabolic signatures in patients with intact PTH using mass spectrometry-based metabolomics. This study was a cross-sectional study. Ultra performance liquid chromatography-Quadrupole Time-of-Flight/mass spectrometry-based metabolic profiling was employed to analyze serum samples from 19 disease controls (DCs) (intact parathyroid hormone [iPTH] 150–300 pg/mL) and 19 secondary hyperparathyroidism (SHPT) patients (iPTH >300 pg/mL) (the training data set) to identify metabolic biomarkers for CKD-MBD. Then, another set of samples including 19 DCs (iPTH 150–300 pg/mL) and 19 SHPT patients (iPTH >300 pg/mL) (the test data set) were used to validate the potential biomarkers identified.

Metabolic profiling analyses revealed different patterns of endogenous metabolites between the SHPT and the DC groups. A total of 32 unique metabolites were identified and 30 metabolites were elevated in the iPTH compared with control serum pools. Cytidine and l-phenylalanine were downregulated in the SHPT patients. The metabolic signatures identified were assessed respectively by an internal 10-fold cross validation with an accuracy of 91.4% and an external validation with an accuracy of 71.1%, a sensitivity of 73.7%, and a specificity of 68.4%.

Mass spectrometry-based metabolomic analyses for SHPT patients promises immense potential for early diagnosis and therapy monitoring. Our results indicated that the onset of CKD-MBD is associated with pathway changes of protein synthesis and metabolism, amino acid metabolism, energy metabolism, and steroid hormone metabolism, with obvious promise for better understanding the pathogenesis of this disease. Several metabolic biomarkers were identified, which warrant further development.

## INTRODUCTION

Chronic kidney disease (CKD) is a condition characterized by a gradual loss of kidney function over time. Early detection can help to prevent the progression of kidney disease to kidney failure. Unfortunately, the mechanisms underlying it have not been well characterized. Small-molecule metabolites have an important role in biological system^1,2^ and represent attractive biomarkers to understand CKD. In the present study, we employed mass spectrometry-based metabolomics to analyze small molecules in patient serum using a ultra performance liquid chromatography (UPLC)-Quadrupole Time-of-Flight (Q-TOF)/mass spectrometry (MS) system.^3,4^ The molecule signatures identified were validated using a chemometric approach to assess their abilities to differentiate the secondary hyperparathyroidism (SHPT) patients from the controls.

## METHODS

### Participants

Written consents were collected from all the patients who participated in this study.^5^ The protocol of the study and the procedures designed for sample collection were reviewed and approved by the ethical committee of The Second Military Medical University, Shanghai, China. Venous blood samples were obtained from patients recruited at the Department of Nephrology, Changhai Hospital of the Second Military Medical University. About 76 uremic patients, who needed maintenance peritoneal dialysis (MPD), were recruited. Among them, 38 patient samples were used for the training data set and the other 38 samples for the testing data set. Based on the level of classical clinical marker, intact parathyroid hormone (iPTH), 2 groups of patients were designed for each data set including the low parathyroid hormone (PTH) group and the high-PTH group. The low PTH group had 19 patients with an iPTH level between 150 and 300 pg/mL, and the high-PTH group had 19 patients with an iPTH level >300 pg/mL. Patient characteristics are shown in Tables 1 and 2.

**TABLE 1:**
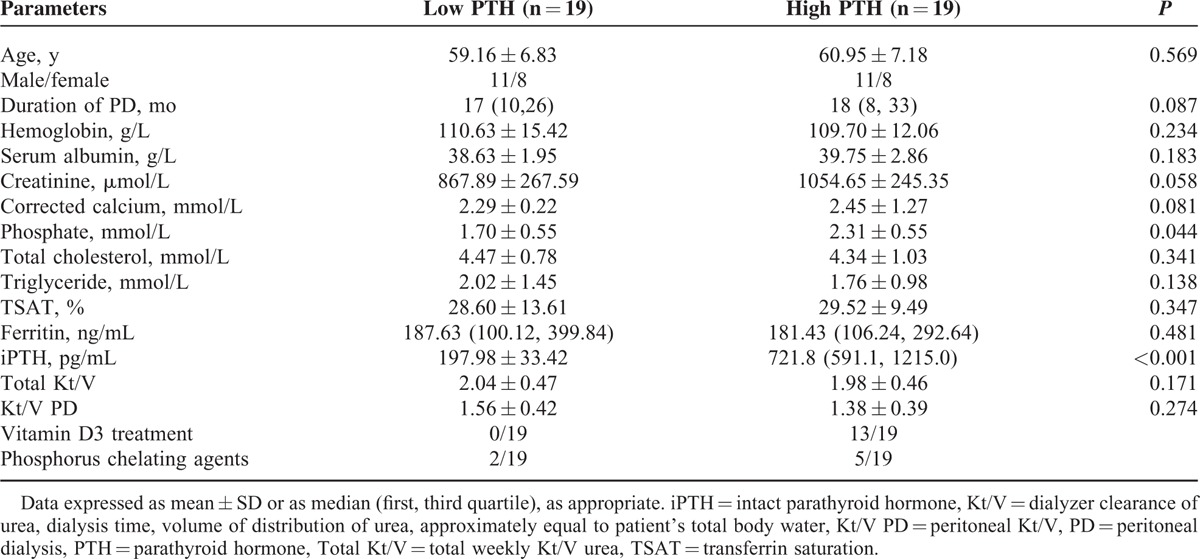
Demographic Description of PD Patients for the Training Set

**TABLE 2:**
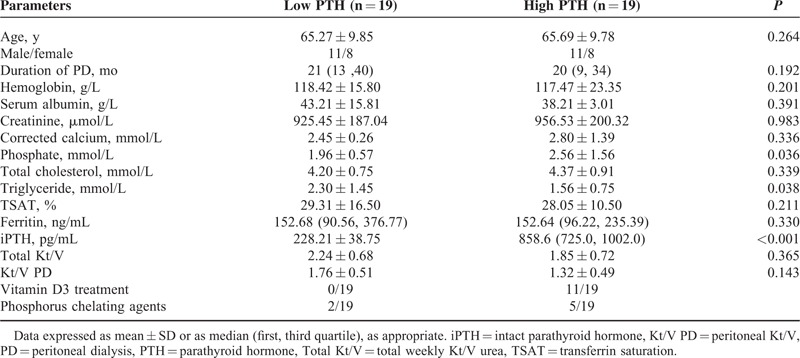
Demographic Descriptions of PD Patients for the Test Set

### Sample Collection and Preparation

Blood sample collection from the patient was done on the same day. Parameters including sex, age, duration of peritoneal dialysis, hemoglobin, serum albumin, blood urea nitrogen, creatinine, corrected calcium, phosphate, total cholesterol, triglyceride, and iPTH levels from each patient were recorded at the sampling. Patients associated with diabetes and hypertension were noted for record. Venous blood was collected into a 5-mL Vacutainer tube containing chelating agent ethylene diamine tetraacetic acid. The tube was centrifuged at 3000 rpm for 15 minutes. The supernatant (serum sample) was aliquoted and stored at −80°C until analysis. No sample went >2 freeze−thaw cycles prior to an liquid chromatography (LC)-MS analysis.

The serum sample (100 μL) was thawed at 4°C followed by the addition of 400 μL methanol/acetonitrile (1:1). The mixture was then vortexed vigorously for 30 seconds followed by centrifugation at 14,000*g* for 15 minutes at 4°C. The supernatant (50 μL) was transferred to an autosampler vial, and an aliquot of 4 μL was injected for LC-MS analysis.

### UPLC-MS Analysis

In this study, an Agilent 1290 Infinity LC system configured with an Agilent 6530 Accurate-Mass Q-TOF mass spectrometer (Agilent, Santa Clara, CA) was used to perform the LC-MS analysis. An ACQUITY UPLC HSS T3 column (2.1  × 100 mm, 1.8 μm; Waters, Milford, MA) was applied to separate serum samples at 45°C with a flow rate of 0.4 mL/min. The mobile phase A had 0.1% formic acid, whereas the mobile phase B had ACN modified with 0.1% formic acid. The gradient program was used as follows: 100% A at 0–2 minutes, 100%–85% A at 2–10 minutes, 85%–70% A at 10–14 minutes, 70%–5% A at 14–17 minutes, 5% A at 17–19 minutes, 5%–100%A at 19–20 minutes, and followed by 5-min column re-equilibration.

An Agilent 6530 Accurate Mass Q-TOF mass spectrometer (Agilent) was adapted to detect ion peaks, and the detection was operated at a negative ion mode. The cone gas was nitrogen with a flow rate of 11 L/h. The following detection parameters were used: fragment voltage, 120 V; capillary voltage, 3.5 kV; gas temperature, 350°C; source temperature, 120°C. The full MS scan mode was monitored at the mass range of 50–1000 m/z. In the analyzing process, 10 mM purine (m/z 121.0508) and 2 mM hexakis phosphazinen (m/z 922.0097) were applied as the internal standards to guarantee mass accuracy and reproducibility. The centroid data were collected from the instrument. Subsequently, an MS/MS experiment was performed, and the experiment parameters were set as follows: MS spectrum acquisition rate, 2 spectra/s; MS/MS spectrum acquisition rate, 0.5 spectra/s; medium isolation window, 4 m/z; collision energy, 20 V.

### Data Analysis

After exclusion of isotope interferences, alignment, and normalization, the resulting data including chromatographic retention times (RT), m/z pairs, sample names, and the normalized ion intensities, were treated with the nearest neighbor imputation approach to deal with the missing data. For a multiple comparison, the false discovery rate (FDR) was used to select the significant variables.^6^ The FDR is designed to control the expected proportion of incorrectly rejected null hypotheses. The variables with q false discovery rate < 0.05 were considered to be significant variables. The partial least squares-discriminant analysis approach was used to differentiate the high-PTH group from the low PTH group.^7^ The internal 10-fold cross validation and external validation were used to test the model. All analyses were carried out with the software package SAS version 9.3 (SAS Institute, Cary, NC) and Comprehensive R Archive Network at http://CRAN.R-project.org/package=caret, USA caret package.^8^

## RESULTS

### Clinical Characteristics of Participants

About 76 uremic patients who were being under MPD therapy were recruited in the study. Demographic characteristics were demonstrated in Tables 1 and 2.

### Statistical Results

**FIGURE 1:**
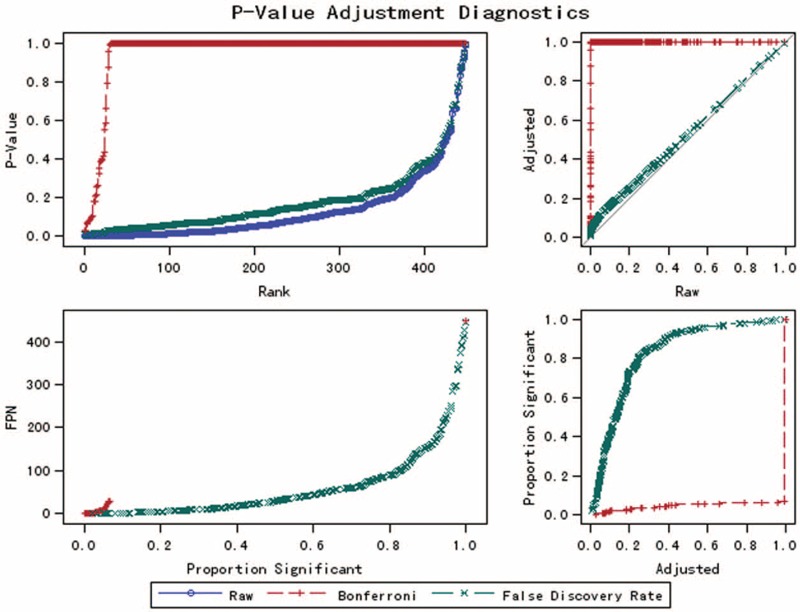
Adjusted *P* value plots. FPN = the number of false positives.

As shown in Figure 1, compared with the raw *P* values, the FDR controlling procedure had a relatively low power and a low type I error rate. However, compared with the familywise error rate procedures, for example, Bonferroni correction, the FDR procedures have greater statistical power. The top 32 variables with the largest values of FDR (*P* < 0.05) were selected for the potential biomarkers (as shown in Table 3). Using a 10-fold cross validation, the accuracy of the classification is about 91.4% when the significant variables were selected by the FDR procedure. The results of the external validation were as follows: accuracy, 71.1%; sensitivity, 73.7%; specificity, 68.4%.

**TABLE 3:**
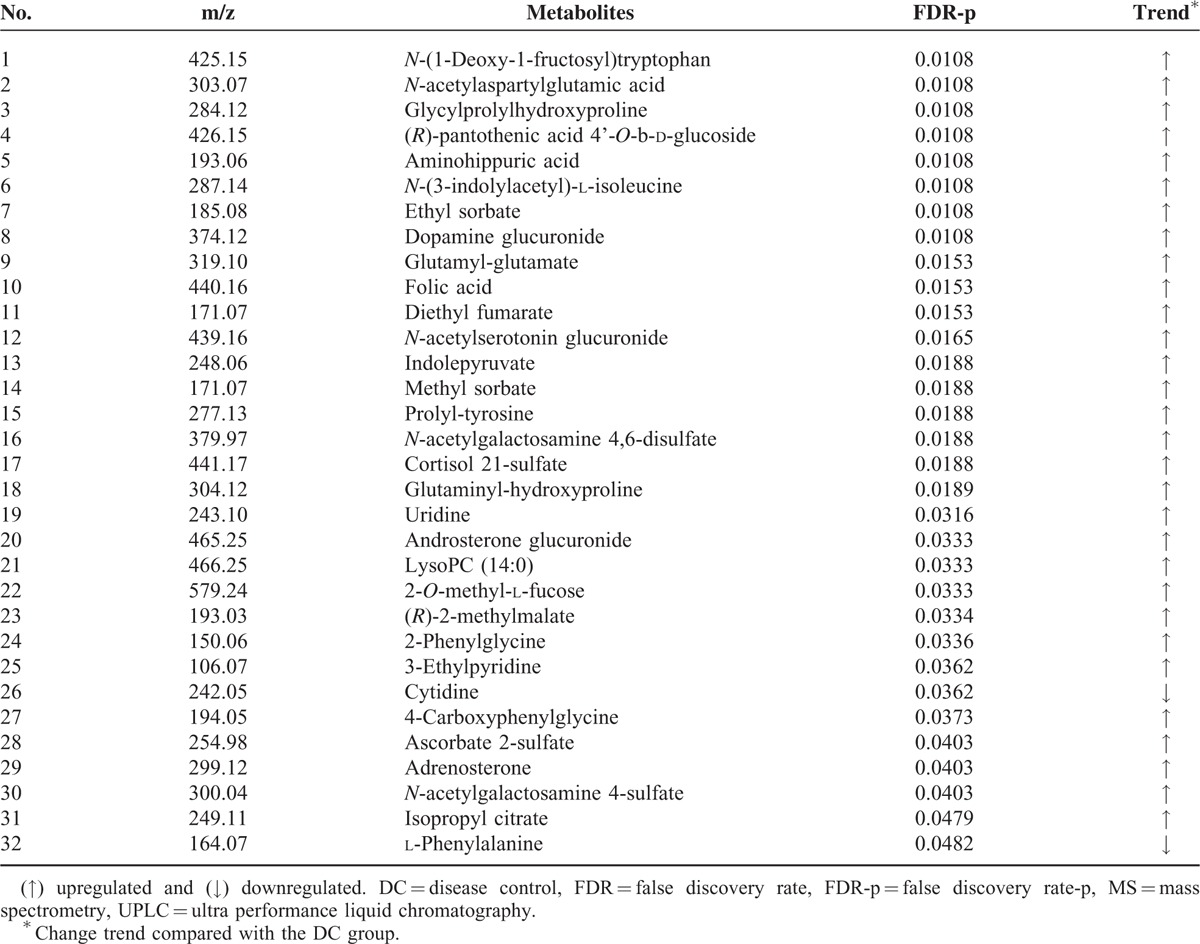
Identification of Potential Differential Metabolites in Serum by UPLC–MS Analysis

## DISCUSSION

Using UPLC-Q-TOF/MS-based metabolic profiling, we found that the metabolic pathway patterns were shifted for the high-PTH patients as compared with the low PTH group. Through metabolomic analysis, 32 metabolites with the largest values of FDR (*P* < 0.05) are selected for potential biomarkers. Interestingly, most of these metabolites identified are not primary metabolites. Instead, these are metabolic substrates and/or products, which are different from the findings of our previous metabolomic analysis.^9^ The metabolic pathway changes identified in this report suggest that other unidentified metabolic pathways are involved in the onset of CKD- mineral and bone disorder (MBD) in addition to the established metabolic pathways of hyperphosphatemia, hypercalcemia, hyperparathyroidism, and active vitamin D deficiency.^10^

### Protein Synthesis and Metabolism

The upregulated metabolites identified in the study with high value of FDR (*P* < 0.05) includes *N*-(1-Deoxy-1-fructosyl)-tryptophan, dopamine glucuronide, *N*-acetylserotonin glucuronide, and prolyl-tyrosine. These are either metabolic substrates or products of other metabolites, which are involved in the protein synthesis and metabolism. For instance, l-phenylalanine is an essential amino acid needed for the synthesis of protein, melanin, and tyrosine. Tyrosine is a precursor of catecholamines including dopamines.^11^ The decreased level of l-phenylalanine combined with the elevated level of prolyl-tyrosine in serum suggests that the metabolic pathway of phenylalanine to tyrosine was downregulated in the SHPT patients. As a result of this, the elevated level of tyrosine activates the biosynthetic pathway of dopamine. This fits our observation that metabolites including prolyl-tyrosine, dopamine, and glucuronide were upregulated and metabolite l-phenylalanine was downregulated in the serum of high-PTH patients. Tryptophan, another essential amino acid, plays an important role in the process of protein synthesis and metabolic network adjustment. Serotonin can be produced by tryptophan through the serotonin pathways. The elevated level of *N*-(1-Deoxy-1-fructosyl)tryptophan and *N*-acetylserotonin glucuronide in high-PTH patients may suggest that the serotonin pathway involves in the onset of CKD-MBD. In addition, previous study has reported that phenylalanine and tryptophan play an important role in calcium homeostasis by activating calcium receptor and dependent suppression of PTH secretion.^12^ Basically, disturbance of calcium homeostasis is a typical feature of CKD-MBD. It was also reported that the elevated level of dopamine is associated with the upregulated phosphate level. Hyperphosphatemia is another symptom of CKD-MBD. Thus, the increased level of dopamine glucuronide observed in our study may be a potential biomarker involved in the onset of CKD-MBD.^13^ It has been reported that serotonin increases the PTH effect on activating protein-1 activity, particularly on the regulation of collagenase expression.^14^ The increased *N*-acetylserotonin glucuronide in high-PTH patients may indicate that serotonin may be involved in the development of CKD-MBD.

### Amino Acid Metabolism

Glycylprolylhydroxyproline, glutaminyl-hydroxyproline, aminohippuric acid, 2-phenylglycine, 4-carboxyphenylglycine, and *N*-(3-Indolylacetyl)- l-isoleucine, as found upregulated in high-PTH patients, are metabolic products of hydroxyproline, glycine, and isoleucine, respectively. Hydroxyproline is reported as a serum bone biomarker. The increased level of hydroxyproline in high-PTH patients may be related to high turnover renal osteodystrophy (ROD).^15^ ROD is a well-known bone pathology, which appears in patients with chronic renal failure, especially in predialysis and dialysis patients. ROD might be another characteristic of CKD-MBD. Thus, hydroxyproline may be a potential marker for the onset of CKD-MBD. In coincidence with previous literature, PTH can inhibit the transcellular transport of aminohippuric acid by activating protein kinase C pathway^16^; the accumulated aminohippuric acid was also found from the serum from the high-PTH group, which is corresponding to the increased level of aminohippurate in high-PTH patients.

### Energy Metabolism

Our study revealed that diethyl fumarate, (R)-2-methylmalate, isopropyl citrate, glutamyl-glutamate, and *N*-acetylaspartylglutamic acid were all increased in the high-PTH group as compared with the low-PTH group. As reported, these 4 metabolites are substrates of the TCA cycle intermediates. Glutamyl-glutamate and *N*-acetylaspartylglutamic acid are substrates of glutamate, which are synthesized by another TCA cycle intermediate, oxaloacetate. Therefore, the abnormal metabolism of TCA cycle and malfunction of mitochondria may be associated with the enhanced level of iPTH. Mitochondria are a very important cellular organelle, which supply energy for cellular activity via oxidative phosphorylation.^11^ We reason that the development of CKD-MBD may be due to a disrupted energy metabolism. However, numerous metabolomic studies found the TCA cycle intermediates are involved in different kinds of diseases. Therefore, those metabolites may be less specific when being used as biomarkers in the CKD-MBD study. In addition, metabolites including *N*-acetylgalactosamine 4-sulfate, *N*-acetylgalactosamine 4,6-disulfate, and 2-*O*-methyl-l-fucose were also found upregulated in high-PTH patients. These metabolites are structural components of glycoproteins. Therefore, we reason that upregulation of these metabolites in the high-PTH group may indicate that glycoprotein catabolism is enhanced with an increased iPTH level. Metabolite galactosamine, often acetylated in nature and a product of glycolysis, can induce endoplasmic reticulum (ER) stress.^17^ Thus, we believed the metabolite disruption would be the link to ER stress that took place in the endothelium and which had been recognized as a key issue in vascular injury.

### Steroid Hormone Metabolism

In the present study, we also noted that there was a significant increase of androsterone and its glucuronide conjugate form in the serum of high-PTH patients. Androsterone, one of the sex hormones, is a product of oxidative metabolism of cholesterol. Androsterone plays an important role in human health and well-being for both men and women. This metabolite influences many aspects of human physiological functions, activities, and behaviors. Disturbance in sex hormone metabolism is an important feature of cancer pathophysiology. A previous study has reported that abnormal changes of sex hormone were associated with lung adenocarcinoma^18^ and adrenocortical carcinoma.^19^ It was also related with the vascular dysfunction by enhancing the release of nitrogen monoxide.^20^ Therefore, sex hormones might also be a factor, which contributes to the development of CKD-MBD.

The levels of the following metabolites were also found being changed in high-PTH group as compared with low-PTH groups. These are nucleosides including uridine and cytidine, lipids including ethyl sorbate, methyl sorbate, and lysoPC (14:0), vitamins including (*R*)-pantothenic acid 4’-*O*-b-d-glucoside and ascorbate 2-sulfate. Interestingly, we found the level of folic acid was increased in high-PTH patients. Folic acid is an essential ingredient for body growth. Bone growth is part of the growing process. Thus, folate may play a role in bone growth. The detailed mechanism is not yet well understood and warrant further study.^21^

The strength of this study is that LC-MS-based metabolic profiling was first used to analyze metabolites in MPD patients with different levels of iPTH to identify metabolic signatures related to CKD-MBD. In this exploratory endeavor, we identified different metabolic patterns in the high-PTH group as compared with the low-PTH group. The limitations of the study include a limited number of participants, the clinical markers used for patient grouping, and the limited health controls.

## CONCLUSIONS

In summary, with the highly sensitive metabolomic technique, our study has observed significant metabolic signature shifts related with the onset of CKD-MBD. Our findings suggest that in addition to the complex mineral and hormone changes such as hyperphosphatemia, hypercalcemia, hyperparathyroidism, and active vitamin D deficiency, other unidentified metabolic pathways and mechanisms may also play important roles in contributing to the development of CKD-MBD. These metabolites identified are potential targets for better prognosis, risk assessment of complications in uremic patients, and exploring strategies for the improvement of compositions of peritoneal dialysis fluid or synthesis of new drugs. It will benefit the treatment of uremic patients.
